# Controlling pH in shake flasks using polymer-based controlled-release discs with pre-determined release kinetics

**DOI:** 10.1186/1472-6750-11-25

**Published:** 2011-03-23

**Authors:** Marco Scheidle, Barbara Dittrich, Johannes Klinger, Hideo Ikeda, Doris Klee, Jochen Büchs

**Affiliations:** 1 Aachener Verfahrenstechnik - Biochemical Engineering, RWTH Aachen University, Sammelbau Biologie, Worringer Weg 1, D-52074 Aachen, Germany; 2 Textile Chemistry and Macromolecular Chemistry, RWTH Aachen University, Pauwelsstr. 8, 52074 Aachen, Germany

## Abstract

**Background:**

There are significant differences in the culture conditions between small-scale screenings and large-scale fermentation processes. Production processes are usually conducted in fed-batch cultivation mode with active pH-monitoring and control. In contrast, screening experiments in shake flasks are usually conducted in batch mode without active pH-control, but with high buffer concentrations to prevent excessive pH-drifts. These differences make it difficult to compare results from screening experiments and laboratory and technical scale cultivations and, thus, complicate rational process development. In particular, the pH-value plays an important role in fermentation processes due to the narrow physiological or optimal pH-range of microorganisms. To reduce the differences between the scales and to establish a pH-control in shake flasks, a newly developed easy to use polymer-based controlled-release system is presented in this paper. This system consists of bio-compatible silicone discs embedding the alkaline reagent Na_2_CO_3_. Since the sodium carbonate is gradually released from the discs in pre-determined kinetics, it will ultimately compensate the decrease in pH caused by the biological activity of microorganisms.

**Results:**

The controlled-release discs presented here were successfully used to cultivate *E. coli *K12 and *E. coli *BL21 pRSET eYFP-IL6 in mineral media with glucose and glycerol as carbon (C) sources, respectively. With glucose as the C-source it was possible to reduce the required buffer concentration in shake flask cultures by 50%. Moreover, with glycerol as the C-source, no buffer was needed at all.

**Conclusions:**

These novel polymer-based controlled-release discs allowed buffer concentrations in shake flask media to be substantially reduced or omitted, while the pH remains in the physiological range of the microorganisms during the whole cultivation time. Therefore, the controlled-release discs allow a better control of the pH, than merely using high buffer concentrations. The conditions applied here, i.e. with significantly reduced buffer concentrations, enhance the comparability of the culture conditions used in screening experiments and large-scale fermentation processes.

## Background

Shaken bioreactors are the most important reaction vessels for screening production strains and for process development. Therefore, several thousand shake flask experiments are performed each year [1,2].

Biotechnological processes are usually conducted in fed-batch cultivation mode with active pH-monitoring and control. In contrast, shake flask experiments are usually conducted in batch mode without active pH-control, but with high initial buffer concentrations to prevent excessive pH-drifts during the cultivation [3,4]. High buffer and substrate concentrations in small-scale cultivation media lead to low water activity and high osmolarity. These conditions may inhibit the growth of microorganisms. For example, since the optimal osmolarity of a medium to cultivate *Escherichia coli is *approximately 0.3 Osmol/L, increasing or decreasing the osmolarity results in reduced bacterial growth rates [5,6]. Consequently, the application of high buffer and substrate concentrations in the screening for optimal production strains might handicap microorganisms with high potential but low osmotolerance. Therefore, a new system which would avoid the use of buffer in screening experiments would enhance the output of screening projects.

For fed-batch cultivation in shake flasks and microtiter plates, an enzyme-controlled glucose auto-delivery system was published recently [7,8]. Furthermore, a polymer-based controlled-release system for fed-batch cultivation of microorganisms in shake flasks without the need for additional equipment or enzymes was developed by Jeude et al. [3]. This controlled-release system contains a silicone elastomer (polydimethylsiloxane) matrix in which glucose crystals are encased. Various applications benefit from this system allowing the controlled-release of glucose in shake flasks (disc-shaped form freely suspended in culture media) [3] or in microtiter plates (suspended or immobilized at the bottom of each well) [9-11]. This controlled-release of glucose in fed-batch cultivations in shake flasks thereby allows the user to reduce the initial substrate concentration in the medium.

The pH-value is an important parameter for cultivating microorganisms. The best pH-range for cultivating e.g. *Escherichia coli *is 6.5-7.5, and varies with temperature [12,13]. The metabolic activity of the cultivated microorganisms influences the pH-value of the surrounding medium in different ways. As microorganisms cultivated in mineral media with glucose or glycerol as carbon source and ammonium salt as nitrogen source, produce one proton [H^+^] per consumed molecule of ammonium [13-16], the pH of the medium decreases during the cultivation. Furthermore, under oxygen-limiting conditions and in the overflow metabolism, *E. coli *produce acetate. This also decreases the pH-value. Once the glucose is depleted and the pH in the medium is not too low for growth of the microorganisms, the accumulated acetate is consumed as a second carbon source and the pH subsequently increases [17,18]. Moreover, metabolically generated bicarbonate ions accumulate in the medium and decrease the pH-value [15]. Therefore, the pH-value changes significantly during fermentations. Consequently, a pH-control is absolutely vital to maintain physiological pH values during microbial cultivation [19].

One of the most important differences between small- and large-scale is the control of the pH of the medium. Weuster-Botz et al. presented a system for pH-control in shake flasks that applies pH-probes, pumps, storage vessels for pH controlling agents and other equipment [19,20]. However, as this system is complex, it is impractical for high-throughput applications.

**Figure 1:**
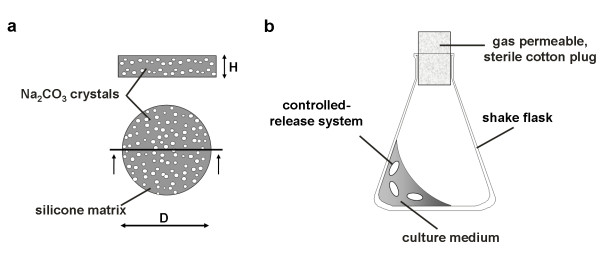
**Controlled release discs for pH-control in shake flasks**. a: Cut and top view of a controlled-release disc. b: Shake flask filled with culture medium and controlled-release discs (Figure adapted from Jeude et al. 2006 [3]).

An easy-to-use polymer-based controlled-release system for keeping the pH in shake flasks in a reasonable range is presented in this paper. It is based on the fed-batch system described by Jeude et al. [3]. This newly developed system has a disc-shaped form and consists of a biocompatible silicone matrix (polydimethylsiloxane) in which the alkaline reagent sodium carbonate is embedded (Figure 1). This sodium carbonate is then gradually released from the discs in pre-determined kinetics and thus increases the pH-value of the medium. The focus of this research study is to reduce the buffer concentrations in cultivation media in order to decrease the osmolarity so that they can be better compared to large-scale media. The pH values during the fermentation should stay in the physiological range of the microorganisms. To demonstrate the applicability of these controlled-release discs in shake flask cultures, *Escherichia coli *K12 and *Escherichia coli *BL21 pRSET eYFP-IL6 were used as model microorganisms. The strain *E. coli *BL21 pRSET eYFP-IL6 produces a fusion protein of enhanced Yellow Fluorescent Protein (YFP) and interleukin-6. The two strains were cultivated in media containing glycerol or glucose as carbon source, respectively.

## Methods

### Organisms

*E. coli *K12 and *E. coli *BL21 pRSET eYFP-IL6 [21] were used as model microorganisms. Stock solutions were maintained in glycerol at -80°C in LB medium. *E. coli *BL21 pRSET eYFP-IL6 cultures additionally contained 100 μg/mL ampicilin.

### Media and Solutions

Modified Wilms & Reuss synthetic medium (henceforth referred to as Wilms-MOPS medium) was used for *E. coli *cultivations [18,22]. The medium consists of 20 g/L glucose or 20 g/L glycerol; 5 g/L (NH_4_)_2_SO_4_; 0.5 g/L NH_4_Cl; 3 g/L K_2_HPO_4_; 2 g/L Na_2_SO_4_; 0.5 g/L MgSO_4_·7H_2_O; 41.85 g/L (0.2 M) 3-(N-morpholino)-propanesulfonic acid (MOPS); 0.01 g/L thiamine hydrochloride; 1 mL/L trace element solution (0.54 g/L ZnSO_4_·7H_2_O; 0.48 g/L CuSO_4_·5H_2_O; 0.3 g/L MnSO_4_·H_2_O; 0.54 g/L CoCl_2_·6H_2_O; 41.76 g/L FeCl_3_·6H_2_O; 1.98 g/L CaCl_2_·2H_2_O; 33.39 g/L Na_2_EDTA (Titriplex III). For cultivations with *E. coli *BL21 pRSET eYFP-IL6 0.1 g/L ampicillin were added. The pH was adjusted to 7.5 with NaOH. The typical MOPS buffer concentration of 0.2 M was used for precultures and for reference cultivations with glucose and glycerol, respectively. For other experiments different MOPS buffer concentrations and different initial pH-values were used and are mentioned in the respective experiment description.

### Cultivation

For online monitoring of oxygen transfer rates (OTR) of all cultures a Respiration Activity Monitoring System (RAMOS) device, fabricated in-house and previously described by Anderlei et al. [23,24], was used. A commercial version of this device is available from HiTec Zang GmbH (Herzogenrath, Germany) or Kühner AG (Birsfelden, Switzerland). The following cultivation parameters were applied: 350 rpm shaking frequency, 50 mm shaking diameter, 10 mL filling volume in 250 mL RAMOS flasks. Precultures and main cultures were cultivated in Wilms-MOPS synthetic medium. For inoculating main cultures, fresh precultures (grown to an OTR of ca. 0.05 mol/L/h) were centrifuged, washed in 5 mL fresh medium, centrifuged again and the pellet was finally resuspended in 5 mL medium. Then optical densities (OD) were measured and used for calculating the required inoculation volume for initial OD values of 0.5 for each experiment. The initial pH-values of the main cultures were set to 7 or 7.5 as indicated in the respective experiment descriptions.

### The polymer-based controlled-release discs containing Na_2_CO_3_

To keep the pH-value of a cultivation in a narrow range without using high buffer concentrations, a polymer-based controlled-release system with embedded Na_2_CO_3 _was developed. In this work disc-shaped controlled-release systems were used. The controlled-release systems are produced as foils during the manufacturing process. Then discs are cut out from the foil. These deliver the preferred mostly linear release kinetics of the sodium carbonate.

The release system was composed of commercially available solvent-free two-component (called component A and B by the manufacturer) silicone Sylgard™184 as well as the catalyst Syl-off™ 4000 (Dow Corning, Wiesbaden, Germany) in a concentration of 0.1% (w/w). The platinum catalyst Syl-off™ 4000 accelerates the cross-linking of the two components. The ratio between the two components A and B was 10:1 as recommended by the manufacturer. Analytical grade Na_2_CO_3 _was supplied by Sigma Aldrich (Crailsheim, Germany). The Na_2_CO_3 _was milled with a vibration micromill (Spartan™, Fritsch, Idar-Oberstein, Germany) in a high-grade steel mortar and then sieved through test sieves (Fritsch, Idar-Oberstein, Germany). The fraction with particle sizes ranging from 20 to 50 μm was used. First, a mixture consisting of component A of Sylgard™184, Na_2_CO_3 _(30% (w/w)) and catalyst was weighed and degassed in a desiccator in a 30 mbar vacuum for 0.5 h. Then, component B was added. The finished mixture was subsequently casted as a thin foil onto a glass plate with a casting knife (gap 1.1 mm) and then cross-linked at 50°C in a convection oven for 3 h. Then discs having a diameter of 15 mm were stamped out (surface area 405.26 mm^2^) and applied for the experiments. The disc-shaped controlled-release systems then gradually release the embedded Na_2_CO_3 _in an aqueous system at a pre-determined rate, so that the medium was alkalized during cultivation, thus counteracting any biological acidification.

The controlled-release discs can not be autoclaved, as the wet environment dissolves the sodium carbonate from the matrix. For the presented experiments the controlled-release discs were sterilized with 70% ethanol and, subsequently, washed with sterile water for one minute. This sterilization method was used directly before the experiments starts. For long term sterilization the controlled-release discs can be treated with gamma rays.

### Measurement of Na_2_CO_3_-release kinetics from the polymer-based controlled-release discs

For measuring the release kinetics of Na_2_CO_3 _from the controlled-release discs, an Na^+^-ion selective electrode was used. With this electrode it is generally possible to determine the concentration of Na^+^-ions within the range from 10^-6 ^mol/L to 1 mol/L. The electrode consists of two half cells, a reference electrode (inLab Reference Pro) and the electrode for the Na^+^-ions. The measuring instrument (SevenMulti) and the electrodes were purchased from MettlerToledo (Gießen, Germany). All salts used were purchased in the highest purity from Sigma Aldrich (Munich, Germany). Destilled water was prepared in a Millipore unit (Millipore, Schwalbach, Gemany). It was used for the preparation of the solutions and kept at a temperature of 20°C. As bridge electrolyte inside the Na^+^-ion selective electrode a 0.1 mol/L NH_4_Cl-solution was applied. As ion adjustment buffer (ISA) an NH_4_Cl/NH_3_-solution was prepared by adding 200 g NH_4_Cl to 50 ml concentrated NH_3_-solution and the volume was filled up with water to a final value of 1 L. For the measurement, the destilled water with ISA-buffer was adjusted to a pH-value of 7 with 1 M KOH. As master solution for the calibration and to store the Na^+^-ion selective half cell, a 0.1 M NaCl solution was prepared. For the calibration sodium chloride was dried 2 h at 120 °C and 5.845 g were weighed and filled up with water to 1 L.

For the calibration comparable conditions such as in the biological experiments were applied (T = 37 °C; agitated with a magnet stirrer). The calibration was done at pH values from 3 to 8 and with sodium carbonate concentrations in the applied concentration range in the experiments of 1*10^-4^; 0.5*10^-4^; 1*10^-3^; 0.5*10^-3^; 1*10^-2^; 0.5*10^-2 ^and 1*10^-1 ^mol/L. The measurement was accomplished with normal sensitivity and in the automatic measuring mode. For measuring the release from the controlled-release discs, 70 ml ISA were employed for each disc. After the experiment the measured controlled-release disc was dried and reweighed for control. In this work controlled-release discs with a diameter (D) of 15 mm and a height (H) of 1.1 mm with a sodium carbonate content of 30% (w/w) were tested.

### Analytical methods

To measure off-line data of the experiments, additional Erlenmeyer flasks were used for sampling. The investigated *E. coli *strains were cultivated in parallel in these flasks under the same conditions as the cultivations in the RAMOS device. For the first sample, depending on the particular experiment, 1 mL or 2 mL of the cultivation broth was withdrawn from the respective flask and the flask was refilled with sterile, purified water. Then, the same respective flask was used for one additional second sample. Each flask was only applied for two samples.

Optical densities were measured at 600 nm (OD_600_) with an Uvikon 922 spectrophotometer (Kontron, Milano, Italy), except for the experiment depicted in Fig. seven Thermo Scientific Genesys 20 spectrophotometer (Waltham, MA, USA) was used. The samples were diluted with fresh medium to measure the OD in the linear range of the photometer and were at least determined twice.

Off-line pH-values were measured with a CyberScan pH 510 (Eutech, Nijkerk, The Netherlands) and with a Titroline alpha device (Schott Instruments, Mainz, Germany). For online measurement of the pH during the cultivations, a combination of a RAMOS device and a fiber optical pH-measuring device (pH-1, Presens, Regensburg, Germany), as described by Scheidle et al., was used [18]. With the fiber optical pH-measuring technique the pH-value was measured every 5 min.

Glycerol, glucose and acetate concentrations were measured with a Dionex HPLC (Dionex, Sunnyvale, USA) with an Organic Acid-Resin 250 × 8 mm (CS-Chromatographie, Langerwehe, Germany) and a Skodex RI-71 detector. Sulphuric acid in a concentration of 5 mM was used as solvent at a flow rate of 0.6 ml/min and a temperature of 60°C.

## Results and Discussion

**Figure 2:**
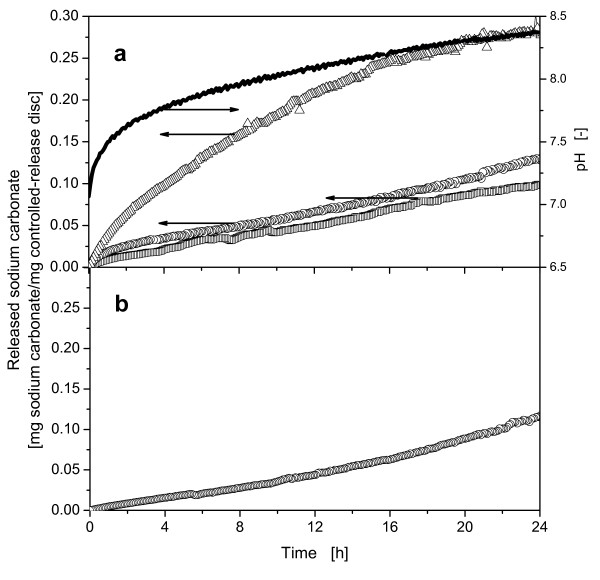
**Release kinetics of different sodium carbonate containing discs a: Release kinetics of discs containing 25% (w/w), 30% (w/w) and 35% (w/w) and pH-course of the disc containing 30% (w/w) sodium carbonate. b: Release kinetics of one 30% sodium carbonate containing disc washed for 30 min in H_2_O**. Released sodium carbonate: 25% (w/w) (□); 30% (w/w) (○); 35% (w/w) (Δ); pH 30% (w/w) (black line); distilled water with ISA buffer; T = 37°C; pH_0 _= 7; controlled-release discs: D = 15 mm; H = 1.1 mm.

In Figure 2a the sodium carbonate release kinetics of controlled-release discs with 25%, 30% and 35% (w/w) sodium carbonate content and, additionally, the pH-course of the disc with 30% (w/w) in the applied buffer are illustrated. During the first 2 h a rapid release of the salt occurred in case of all three applied discs. Subsequently, the sodium carbonate release is nearly linear. The release kinetics of the controlled-release disc with 35% sodium carbonate content was considerably higher than in the other two discs. The controlled-release disc with 30% sodium carbonate exhibits a slightly higher release kinetics compared to the disc with 20%. For the following experiments discs containing 30% (w/w) sodium carbonate were used, because previous growth experiments with *E. coli *demonstrated that these discs have the most suitable release kinetics for the cultivations (data not shown). The pH-course of the 30% sodium carbonate containing controlled-release disc depicts a fast increase during the first 2 h. Then the pH-value increase nearly linear to a value of about 8.4 after 24 h. The rapid release and pH increase at the beginning of the experiment had to be considered in all biological experiments, because microorganisms tend to show lag phases and initially grow slow and, thus, only few protons are produced. Figure 2b depicts the release of one washed 30% (w/w) sodium carbonate containing disc. The disc was washed in H_2_O for 30 min to reduce the initial, fast sodium carbonate release. This method was used for some of the cultivation experiments.

**Figure 3:**
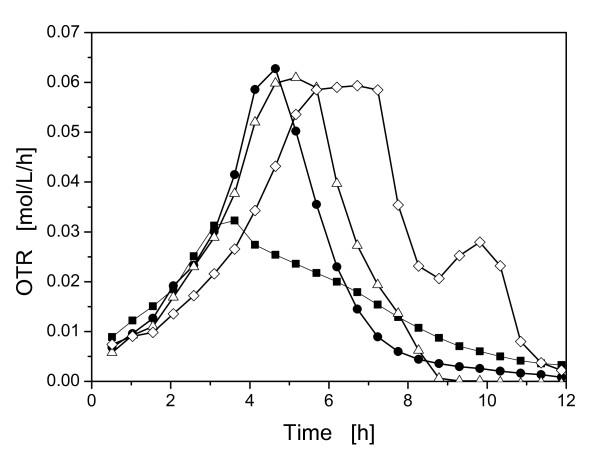
**Oxygen transfer rate (OTR) of *E. coli *BL21 pRSET eYFP-IL6 during growth in Wilms-MOPS medium with different buffer concentrations**. With 0.2 M MOPS buffer (◇), with 0.1 M MOPS buffer (Δ), with 0.05 M MOPS buffer (●), without MOPS buffer (■); experimental conditions: Wilms-MOPS medium with 20 g/L glucose, 37°C; 10 mL filling volume; shaking diameter (d_0_) 50 mm; 350 rpm; OD_600,α _= 0.5; pH_0 _= 7.5.

To investigate the influence of the buffer concentration and, therefore, of the osmotic pressure on the metabolic activity of *E coli *BL21 pRSET eYFP-IL6, cultivations with different MOPS buffer concentrations were conducted. Another main focus of these cultivations was to establish a basis for the following experiments using the controlled-release discs to reduce the used buffer concentrations (Figure 3).

The reference cultivation with 0.2 M MOPS buffer depicted in Figure 3, showed the typical metabolic activity of the microorganisms as described by Scheidle et al. [18]. After about 5.5 h the OTR peaked at a value of ca. 0.06 mol/L/h and then formed a plateau, which indicates oxygen limitation [23]. The second peak, with approximately 0.3 mol/L/h, indicates a second growth phase. Here, acetate is consumed, that was produced during the oxygen limitation. The final pH-value of this cultivation was 7.01.

**Figure 4:**
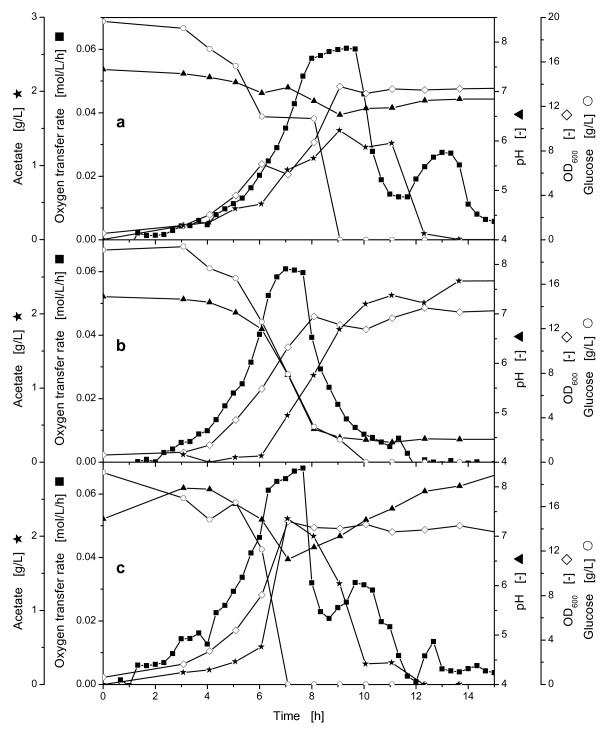
**Comparison of the oxygen transfer rate (OTR), pH and glucose concentration during the cultivation of *E. coli *BL21 pRSET eYFP-IL6 with different buffer concentrations and controlled-release discs**. **a**: with 0.2 M MOPS buffer, **b**: with 0.1 M MOPS buffer, **c**: with 0.1 M MOPS buffer and additional 3 controlled release discs. OTR (■); pH (▲); glucose concentration (○); OD_600 _(◇); acetate concentration (★); experimental conditions: Wilms-MOPS medium with 20 g/L glucose, 37°C; 10 mL filling volume; shaking diameter (d_0_) 50 mm; 350 rpm; OD_600,α _= 0.5; pH_0 _= 7.5; controlled-release discs: D = 15 mm; H = 1.1 mm; 30% (w/w) Na_2_CO_3_.

The cultivation with 0.1 M MOPS buffer peaked about one hour earlier, than the culture with 0.2 M MOPS buffer concentration. A short oxygen limitation can than be recognized before the OTR decreased slowly in a triangle-shaped form. This triangle-shaped form indicates acidic pH-values that impair the metabolic activity of the microorganisms [23] (see Figure 4). Furthermore, the low final pH-value of 4.86 supports this conclusion. The increased specific growth rate (μ_max_) (from 0.44 1/h to 0.53 1/h) between the cultivations with 0.2 M, 0.1 M and the lower MOPS buffer concentrations can be attributed to the higher osmolarity in the medium with 0.2 M MOPS buffer. The Wilms-MOPS media with 0.2 M and 0.1 M MOPS buffer concentration have an osmolarity of ca. 0.65 Osmol/L and ca. 0.48 Osmol/L, respectively. Record et al. described a linear decrease of the specific growth rate with increasing osmolarities of the medium above 0.3 Osmol/L [6]. These authors report that the specific growth rate decreases in minimal medium by half from ca. 0.3 Osmol/L to 1 Osmol/L.

Application of 0.05 M MOPS demonstrates the same OTR curve and μ_max _as the cultivation with 0.1 M MOPS buffer until 4.5 h. Here, the OTR decreased earlier in a triangle-shaped form. The low buffer concentration leads to a fast acidification of the medium to a final pH-value of 3.99 and, therefore, to impaired metabolic activity.

The cultivation without any buffer shows the same metabolic activity and μ_max _as the culture with 0.1 M MOPS buffer until after 3.5 h the OTR peaks and the OTR slightly decreases. Without buffer, the culture acidifies even earlier and the growth is hampered due to the suboptimal final pH-value of 3.57.

These cultivations clearly demonstrate the positive influence of reduced buffer concentrations on the specific growth rates of *E. coil*. However, without the high buffer concentrations the pH decreased very fast to unfavorable values and another method for pH-control is necessary.

To demonstrate the application and the feasibility of the sodium carbonate containing controlled-release discs for controlling the pH in shake flasks, cultivations with *E coli *BL21 pRSET eYFP-IL6 in media with different MOPS buffer concentrations and, in addition, controlled-release discs were performed (Figure 4).

In the reference cultivation with *E. coli*, depicted in Figure 4a, a MOPS buffer in a concentration of 0.2 M was applied. This cultivation showed the same shape depicted in Figure 3.

In the cultivation presented in Figure 4b, 0.1 M MOPS buffer was used to demonstrate the growth of *E coli *BL21 pRSET eYFP-IL6 with a buffer concentration that was 50% less than that in the previous experiment (Figure 4a). After ca. 7.5 h the OTR decreased slowly, until the microorganisms stopped to grow. This slow decrease of the OTR curve indicated a pH-range suboptimal for growth [23]. The pH dropped very fast during the following cultivation until it reached its final value of ca. 4.45. On the basis of the slow decreasing OTR after 7.5 h, the pH-curve and the reduced increase in the OD-values, it could be concluded that the metabolism of *E. coli *pRSET eYFP-IL6, with the applied conditions, is affected at pH-values below 5.8. Here, the pH-value dropped below 5.8, while the OTR decreased and the growth was reduced, although the glucose was not yet depleted. Even though the carbon source glucose was completely exhausted at ca. 10 h, the previously produced acetate was not consumed and no metabolic activity could be observed; this means that the very low pH in the medium completely hampered the activity of the microorganisms.

To demonstrate the applicability of the controlled-release of sodium carbonate for controlling the pH, an experiment with buffer in a concentration of 0.1 M MOPS and three controlled-release discs (30% (w/w) sodium carbonate content) was performed (Figure 4c). Here, the OTR curve followed the same shape as in the reference cultivation with 0.2 M MOPS buffer depicted in Figure 4a. In contrast to the cultivation with 0.1 M MOPS buffer and without controlled-release discs, the OTR peaked twice and the microorganisms completely consumed the previously produced acetate.

Due to the continuously released sodium carbonate (see Figure 2) and the low biomass concentration in the beginning of the cultivation, the pH increased up to a value of 7.98 after ca. 3 h. Thereafter, the pH curve decreased to a value of 6.54 until ca. 6 h cultivation time. This corresponds to the peak of acetate concentration. During this interval, the biomass concentration rose and more protons were produced by the microorganisms than sodium carbonate was released from the controlled-release discs. Once the glucose was depleted and replaced by acetate as carbon source the pH increased. This increase showed a steeper slope than in the cultivation with 0.2 M MOPS buffer, because sodium carbonate is still additionally released continuously from the controlled-release discs.

These experiments proved that the pH-control in shake flasks with controlled-release of sodium carbonate works. However, the release of the sodium carbonate is nearly linear, while the microorganisms are growing exponentially. Therefore, pH-changes during the cultivation are inevitable and the pH can only be controlled in a particular range. To minimize these pH changes the cultivation conditions (e.g. initial pH and buffer concentrations) must be adapted to the particular microorganisms and used cultivation system.

With this technique it was possible to halve the buffer concentration in the Wilms-MOPS medium with 20 g/L glucose and to establish the same growth behavior such as that with the higher reference buffer concentration (0.2 M MOPS buffer) at higher grows rates due to decreased osmotic pressure.

**Figure 5:**
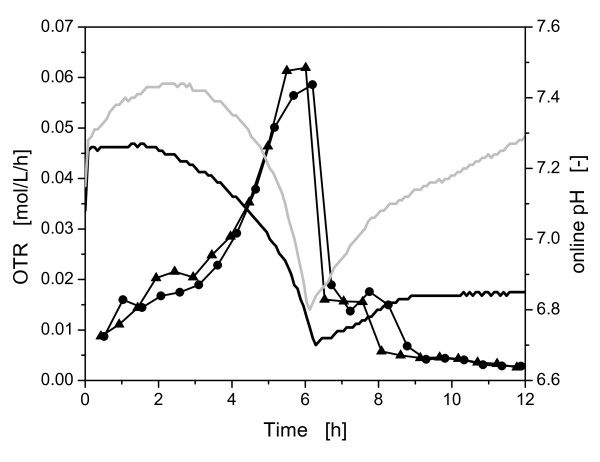
**Comparison of oxygen transfer rate (OTR) and online pH of *E. coli *BL21 pRSET eYFP-IL6 during growth in Wilms-MOPS medium with different buffer concentrations and controlled-release discs**. With 0.2 M MOPS buffer OTR (●) and pH (black line), with 0.1 M MOPS buffer and 3 controlled-release discs, OTR (▲) and pH (grey line). Experimental conditions: Wilms-MOPS medium with 20 g/L glucose, 37°C; 10 mL filling volume; shaking diameter (d_0_) 50 mm; 350 rpm; OD_600,α _= 0.5; pH_0 _= 7.5; controlled-release discs: D = 15 mm; H = 1.1 mm; 30% (w/w) Na_2_CO_3_.

For validating this successful application of the controlled-release discs and to measure the whole pH-course in full detail, *E coli *BL21 pRSET eYFP-IL6 was cultivated in a RAMOS device with integrated online pH measurement [18]. One fermentation with 0.2 M MOPS buffer concentration and one with 0.1 M MOPS and three additional controlled-release discs were conducted (Figure 5). The OTR curves of both cultivations followed nearly the same course. In the beginning of the cultivation with a MOPS buffer concentration of 0.2 M, the pH did not change because of the high buffer capacity of the medium and the relatively low metabolic activity of the microorganisms. During the exponential growth phase of the microorganisms the pH decreased. Subsequently, the pH increased during the time when acetate was consumed.

The pH of the culture with 0.1 M MOPS buffer and three controlled-release discs rose in the beginning of the experiment to a maximum value of ca. 7.7 due to the constant release of sodium carbonate (refer to Figure 2). After ca. 2.5 h, the increased microbial activity yielded more protons compared to the amount of sodium carbonate released. Thus, the pH decreased to a minimal value of ca. 6.3 until glucose was depleted from the medium. In the second growth phase until 8 h the pH increased rapidly because of the consumption of acetate and the continuing release of sodium carbonate. The slope of the increasing pH-values is higher than the slope in the cultivation with 0.2 M MOPS buffer during 6.5 h and 8 h. Then a slope change is clearly visible. Here, the sodium carbonate release resulted in still slightly increasing pH-values during the stationary growth phase of the culture with controlled-release discs. This detailed information about the pH-curve could only be detected using the online pH-measurement technique. These results clearly demonstrate that the application of the controlled-release discs was successful for controlling the pH in *E. coli *BL21 pRSET eYFP-IL6 cultures with 20 g/L glucose in Wilms-MOPS medium. The pH-value was not kept at one defined value as, e.g. in an actively pH-controlled fermenter. However, the changes in the pH do not exceed 0.6 pH-units and are retained in the optimal pH-range for the metabolic activity of *E. coli*. Devey specified this optimal range between 6.5 and 7.5 [12]. Furthermore, the buffer concentrations in the medium could be substantially reduced, thus, improving the comparability of small-scale shake flask cultures with large-scale fermentations.

**Figure 6:**
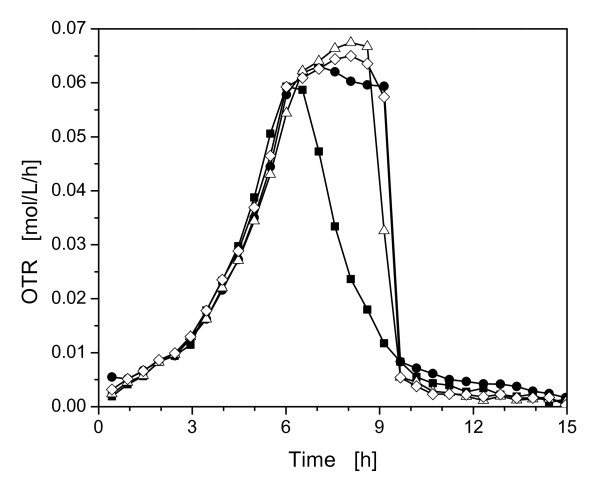
**Influence of different initial pH-values and buffer concentrations on the Oxygen transfer rate (OTR) in *E. coli *K12 cultures in Wilms-MOPS medium using glycerol as carbon source and with and without controlled-release discs**. With 0.2 M MOPS buffer, initial pH 7 (■), 0.2 M MOPS buffer, initial pH 7.5 (◇), with 0.1 M MOPS buffer, initial pH 7 and 3 controlled-release discs (●), with 0.1 M MOPS buffer; initial pH 7.5 and 3 controlled-release discs (Δ). Experimental conditions: Wilms-MOPS medium with 20 g/L glycerol, 37°C; 10 mL filling volume; shaking diameter (d_0_) 50 mm; 350 rpm; OD_600,α _= 0.5; controlled-release discs: D = 15 mm; H = 1.1 mm; 30% (w/w) Na_2_CO_3_, washed for 30 min in H_2_O.

To demonstrate the influence of different initial pH-values in the medium and the application of the sodium carbonate containing controlled-release discs with another strain and carbon source, *Escherichia coli *K12 with Wilms-MOPS medium with 20 g/L glycerol was cultivated in varying buffer concentrations and initial pH values (Figure 6). In this experiment, four cultures were compared - two of which were cultivated at initial pH values of 7, whereby one of these was grown with and the other one without controlled-release discs. The remaining two cultures, in contrast, were cultivated at an initial pH-value of 7.5 and, analogously, one of each grown with and without the controlled-release discs.

Previous experiments with glycerol as carbon source revealed that the growth rate of *E. coli *was lower than with glucose as carbon source. For that reason the sodium carbonate release from untreated controlled-release discs was too fast in the beginning. Consequently, the pH increased rapidly to suboptimal high values (data not shown). Therefore, all experiments with glycerol as carbon source used controlled-release discs, which were washed in water for 30 min. During these 30 min, the very fast release of the Na_2_CO_3 _in the beginning of the release kinetics can be intercepted. The release kinetics of the washed controlled-release discs are described in Figure 2b.

The culture with an initial pH 7 and in the absence of controlled-release discs (squares) showed a triangular OTR-curve indicating that the pH-value became too acidic for normal metabolic activity (see Figure 3). On the contrary, the culture with the higher initial pH-value of 7.5 (diamonds) reached a higher OTR and entered a plateau after about 6 h. At the end of the plateau, the OTR decreased slowly until ca. 9 h and then dropped sharply. The first slight decrease indicated a suboptimal pH-value, before the glycerol was exhausted. The differences in the two cultures with initial pH-values of 7 and 7.5, respectively is dependent on the buffer capacity of the used MOPS buffer. The pK_a_-value of MOPS buffer at 37°C is 6.98 and the pH buffer capacity lies in the range of about ±1 of this pKa-value. Therefore, the experiment with initial pH-value of 7.5 can utilize more of the buffer capacity during the cultivation, than the culture with an initial pH-value of 7.

Using the controlled-release discs at a MOPS buffer concentration of 0.1 M, the culture at an initial pH 7 (circles) depicted a much less impaired metabolic activity compared to the respective culture without sodium carbonate release (squares). Only between ca. 7.5 and 9 h a slightly reduced metabolic activity can be seen in the OTR. The usage of an initial pH of 7.5 and addition of controlled-release discs (triangles) resulted in a preferred OTR curve in this experiment. No impact of suboptimal pH was recognizable. Therefore, an initial pH value of 7.5 is more suitable for *E. coli *K12 under the applied conditions with glycerol as carbon source. The culture can take advantage of the higher utilized buffer capacity of the applied medium than the culture with an initial pH-value of 7. Furthermore, it is evident that the culture parameters (e.g. the initial pH-value and buffer concentration) have to be chosen very carefully to benefit from the pH-control with the polymer-based controlled-release discs. Without the optimal parameters, the pH could drift very fast into too high or too low values for normal metabolic activity of the microorganisms.

To prove the applicability of the controlled-release discs without any addition of buffer in the medium, experiments were performed in Wilms-MOPS medium with 20 g/L glycerol with and without MOPS buffer and controlled-release discs, respectively. A suboptimal initial pH-value of 7 (see discussion Figure 6) was chosen for these cultivations to demonstrate the functionality of the pH-control using controlled-release discs even under suboptimal conditions.

**Figure 7:**
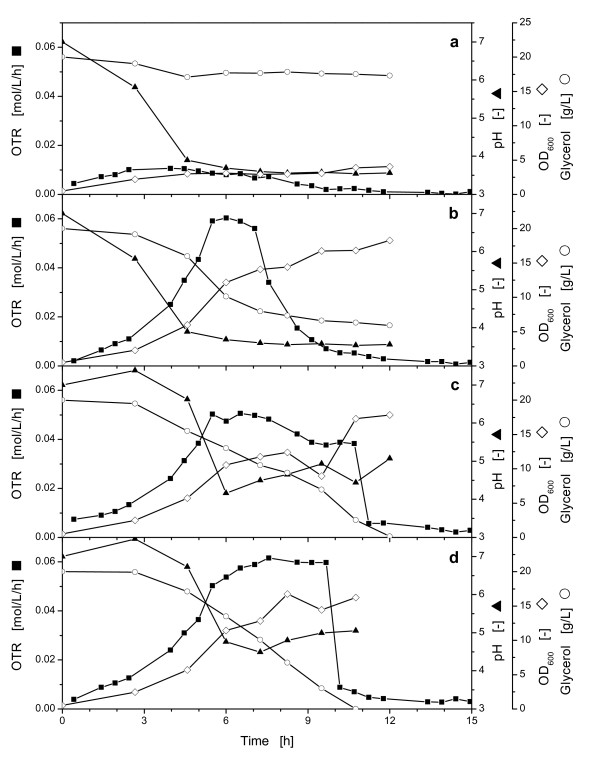
**Comparison of the oxygen transfer rate (OTR), pH and glycerol concentration during the cultivation of *E. coli *K12 in buffered or pH-controlled media using controlled-release discs in shaking flasks**. **a**: without buffer, **b**: with 0.2 M MOPS buffer, **c**: without buffer and additional 3 controlled release discs, **d**: without buffer and additional 4 controlled-release discs. OTR (■); pH (▲); glycerol concentration (○); OD_600 _(♦); Experimental conditions: Wilms-MOPS medium with 20 g/L glycerol, 37°C; 10 mL filling volume; shaking diameter (d_0_) 50 mm; 350 rpm; OD_600,α _= 0.5; pH_0 _= 7; controlled-release discs: D = 15 mm; H = 1.1 mm; 30% (w/w) Na_2_CO_3_, washed for 30 min in H_2_O.

In Figure 7a one cultivation was performed without MOPS buffer. In this experiment the pH decreased rapidly. The pH-value of the medium below 4 hampered the growth of the microorganisms completely. In the second experiment the same conditions were chosen, except for an increased MOPS buffer concentration of 0.2 M (Figure 7b). Applying this standard buffer concentration, the OTR rose to a value of 0.06 mol/L/h in the beginning, followed by a short plateau indicating oxygen limitation and representing the maximum oxygen transfer capacity for this cultivation conditions. Than the OTR decreased in a triangular shape, indicating that the metabolism of the microorganisms is impaired by too low pH [23]. At the end of the cultivation the carbon source glycerol was not depleted which indicates that the metabolism of *E. coli *K12 was indeed hindered by too acidic conditions.

To investigate the influence of the new controlled-release discs, in the third experiment no buffer was used, but three controlled-release discs were added (Figure 7c). The OTR in this experiment displayed a normal growth of the microorganisms until a maximum was reached at 5 h. Thereafter, the OTR decreased slowly until 11 h when the glycerol was depleted. During this time the culture was not oxygen-limited, because the maximum oxygen transfer capacity of 0.06 mol/l/h, as described in Figure 7b, was not reached in this experiment. This gradual decrease in the OTR curve is attributed to the pH-values between 4.2 and 5 which were partially, however, not sufficiently counterbalanced by the Na_2_CO_3 _release from the controlled release discs.

In the fourth experiment no buffer and 4 controlled-release discs were investigated (Figure 7d). In this final experiment, a better metabolic activity could be observed than with three controlled-release discs (Figure 7c). No negative influence of too low pH-values could be observed in the growth and the metabolic activity during this experiment. The complete carbon source glycerol was consumed by the microorganisms. Although the pH-value reached a relatively low value at 4.5, the metabolic activity is sustained, due to the continuous release of the Na_2_CO_3_. The pH-curve depicts the most suited pH range for growth of the utilized microorganisms (*E. coli *K12) on glycerol of these four experiments in a range of ca. 4.5 and 7.4. Therefore, the controlled-release discs enable the user to control the pH without any additional buffer and the osmolarity of the medium was dramatically reduced from 0.78 OsM in the medium with 0.2 M MOPS buffer to 0.44 OsM in the medium without MOPS buffer. The observed changes in the pH-curve are not ideal for growth of *E. coli *K12. However, the controlled-release system enabled metabolic activity and complete consumption of the carbon source even without any buffer.

## Conclusions

The presented polymer-based controlled-release discs embedding sodium carbonate crystals for controlling the pH in shake flasks enabled the successful cultivation of *E. coli *K12 and *E. coli *BL21 pRSET eYFP-IL6 in mineral media with glycerol and glucose as carbon sources, respectively. With the controlled-release discs it is possible to substantially reduce the buffer concentrations or even to omit the buffer in media for shake flask cultures, while the pH-values remain in the physiological range for sustained microbial activity during the whole cultivation. This reduction in buffer concentration leads to reduced osmolarities in the medium. These reduced osmolarities may enhance the growth rates of *E. coli *dramatically, as demonstrated in Figure 3 and described by Record et al. [6].

Large-scale fermentation processes are equipped with an active pH-control. In these processes no buffers are used. The here presented polymer-based controlled-release discs enable comparable cultivation parameters in shake flasks such as in large-scale, while the buffer concentrations are dramatically reduced and the pH is controlled in a narrow range.

In mineral media some of the nutrients, e.g. the phosphate source, function as a buffer. Even in media without any additional buffers, the phosphate concentrations necessary for growth in the media result in a very low buffer capacity. Therefore, when omitting additional buffers while using the controlled-release discs, the medium still has a low buffer capacity. This low buffer capacity can maintain the pH-value to a small degree.

The controlled-release system for fed-batch cultivation in shaken bioreactors developed by Jeude et al. [3] is a self-regulating system. During the lag phase of the microorganisms the released glucose accumulates in the medium [10]. When the biomass concentration increases during the cultivation the accumulated glucose is consumed until the substrate-limited fed-batch phase starts. In the fed-batch phase, the microorganisms directly consume the released glucose. When different cultivation parameters such as various starting biomass concentrations or lag phases occur in parallel cultures (e.g. precultures for screening experiments), then all cultures enter the fed-batch phase at different points in time. When all cultures are in the fed-batch mode, their biomass concentrations and their growth rates are equalized dependent on the glucose release kinetics [10]. In contrast, the pH-control with Na_2_CO_3 _containing polymer-based controlled-release discs presented in this paper is not self-regulating. Here, the Na_2_CO_3 _salt is released and influences the pH-value of the medium, no matter if the microorganisms are growing or not. Additionally, the sodium carbonate is not consumed by the microorganisms such as the glucose in the fed-batch system. Moreover, the nearly linear release of sodium carbonate from the controlled-release discs and the exponential growth leads to inevitable changes in the pH. Therefore, it is very important to ensure optimal growth parameters such as initial pH-values and biomass concentrations while using the newly developed controlled-release discs with pre-defined release kinetics. Especially, the lag phase of a culture has a tremendous effect on the performance of the discs. For example, when the lag phase is too long, the pH increases very fast to suboptimal high values due to the high initial release of the Na_2_CO_3_.

## Outlook

The combination of the controlled-release discs presented in this paper and the glucose delivering controlled-release discs for fed-batch cultivation, described by Jeude et al. [3], has the potential to further increase pH and process control in shake flasks. The defined growth behavior in fed-batch mode being almost linear leads to a defined acidification of the culture. Therefore, the sodium carbonate release for pH-control can be adapted to this defined acidification. The combination of the two controlled-release discs is currently investigated.

In our Laboratory controlled-release systems which enhance the release of alkaline compounds, such as Na_2_CO_3_, in response to reducing pH-values are currently under development. This will lead to balanced pH-regulation in small-scale cultures in the future. With such a pH-responsive controlled release system, it will be possible to cultivate microorganisms showing different lag-times in small-scale shaken bioreactors without any additional buffer, thereby ensuring culture conditions in the optimal pH-range.

## Authors' contributions

MS performed most of the cultivation experiments and prepared the manuscript. BD developed the polymer-based controlled-release discs and measured the release kinetics of the sodium carbonate. JK supported some of the cultivation experiments. HI contributed the cultivation experiment investigating the influence of different buffer concentration on the growth of *E. coli*. DK supervised the preparation of the polymer materials and assisted with data analysis. JB initiated the project, assisted with data analysis and manuscript preparation. All authors approved the final manuscript.
